# Supplementary material for: Saccharomyces boulardii (CNCM I-745) improves intestinal damage in sepsis by remodeling intestinal flora structure
Source: Front Cell Infect Microbiol. 2025 Jul 31;15:1602792. doi: 10.3389/fcimb.2025.1602792 (PMC12350329; doi:10.3389/fcimb.2025.1602792)
Supplement: Supplementary file 1 [file DataSheet1.zip › summary/summary/8_Advanced_analyse/2_PICRUSt2_result/COG/Group.DW4_vs_CW4_vs_BW4_vs_AW4/PICRUSt2_COG_Group.DW4_vs_CW4_vs_BW4_vs_AW4_diff.pdf]

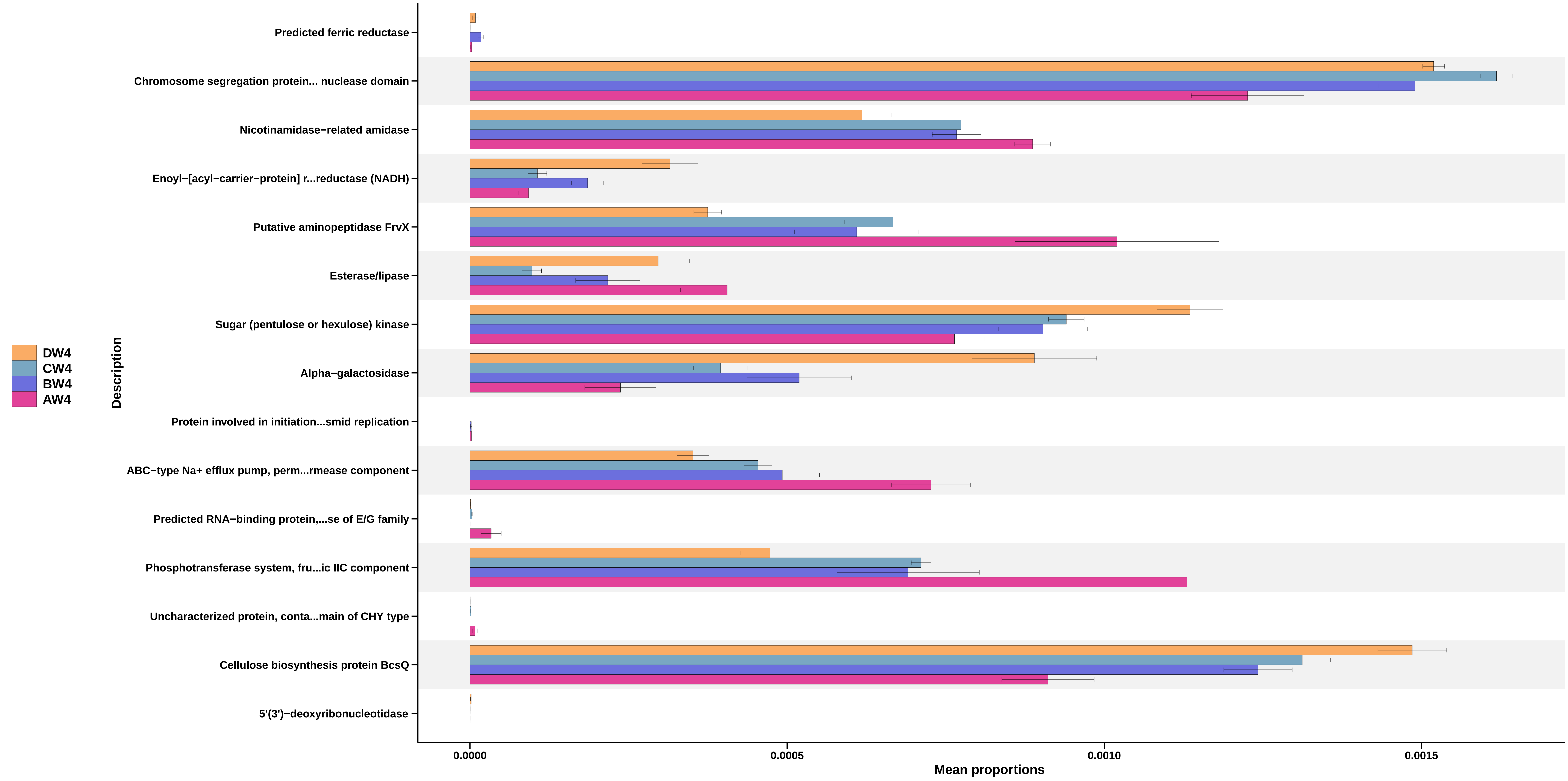
Predicted ferric reductase
Chromosome segregation protein... nuclease domain
Nicotinamidase−related amidase
Enoyl−[acyl−carrier−protein] r...reductase (NADH)
Putative aminopeptidase FrvX
Esterase/lipase
Sugar (pentulose or hexulose) kinase
Alpha−galactosidase
Protein involved in initiation...smid replication
ABC−type Na+ efflux pump, perm...rmease component
Predicted RNA−binding protein,...se of E/G family
Phosphotransferase system, fru...ic IIC component
Uncharacterized protein, conta...main of CHY type
Cellulose biosynthesis protein BcsQ
5'(3')−deoxyribonucleotidase
Description
DW4
CW4
BW4
AW4
0.0000
0.0005
0.0010
0.0015
Mean proportions
